# Neurological Impairment Recovery in Surgically Treated Patients With Nontraumatic Spinal Cord Injury

**DOI:** 10.5435/JAAOSGlobal-D-22-00066

**Published:** 2022-08-11

**Authors:** Sami AlEissa, Faisal Konbaz, Majid Abalkhail, Fahad AlHelal, Lina AlHumaid, Mutlaq AlMutlaq, Faisal AlNaqa, Naila Shaheen

**Affiliations:** From the Department of Orthopedic Surgery, King Abdulaziz Medical City, Central Region (AIEissa, Konbaz, Abalkhail, AIHelal, AINaqa); College of Medicine, King Saud Bin Abdulaziz University for Health Sciences (AIEissa, Konbaz, AIHumaid, AIMutlaq); and the Department of Biostatistics and Bioinformatics, King Abdullah International Medical Research Center (Shaheen).

## Abstract

The incidence of nontraumatic spinal cord injury (NTSCI) is reported to be higher than that of traumatic spinal cord injury in many countries, making it an area of growing significance.

**Objectives:**

With the aging of the global population in coming decades, it is anticipated that the incidence of NTSCIs will increase dramatically. Our aim was to identify and report the causes, patterns, and outcomes of NTSCI in a tertiary care center.

**Methods:**

We have reviewed all adult patients who had a NTSCI and were surgically treated with a minimum follow-up of 12 months postoperatively. Demographic and clinical data were collected. Preoperative and postoperative American Spinal Injury Association (ASIA) impairment scales and past follow-up outcomes were assessed.

**Results:**

Of 164 patients, 95 (58%) had full recovery and reached ASIA E score at their last follow-up while 69 (42%) were not able to achieve full recovery till ASIA E score. Urinary incontinence and/or bowel incontinence on admission, degenerative pathologies, and thoracic injury level were all notable indicators of limited ASIA score improvement at the last follow-up.

**Conclusions:**

Surgically treated NTSCI could result in good neurological recovery with a low complication rate.

Nontraumatic spinal cord injury (NTSCI), as the name implies, refers to damage to the spinal cord that resulted from a cause aside from trauma.^1^ It is a condition with immense functional implications for the individuals involved.^2^ The incidence of NTSCI is difficult to estimate because of its heterogeneous cluster of a wide spectrum of etiologies with varying pathophysiology.^3-5^ The most described NTSCI causes are degenerative disk disease and spinal canal stenosis, tumors, vascular diseases, and inflammatory conditions.^4-6^ The incidence of NTSCI is reported to be higher than that of traumatic spinal cord injury in many countries, making it an area of growing significance.^7,8^ However, the prevalence of studies on NTSCI has not been widely reported and is lacking. Moreover, NTSCI has not been as well studied until relatively recently.^9^ With the aging of the global population in coming decades, as a result, it is anticipated that the incidence of NTSCIs will increase dramatically.^9,10^

Studies have shown that degenerative conditions are the most common cause of NTSCI. Most of these studies were conducted in Europe and North America. A general belief is that there is regional variability in the most common cause of NTSCI, and the data from the Middle East and North Africa region are lacking. The goal of our study was to report the causes, patterns, and outcomes of NTSCI in Saudi Arabia, hoping to fill the gap of knowledge related to this particular topic in the Middle East and North Africa region.

## Methods

### Study Design and Population

This is a retrospective cohort study that involves all patients who had a nontraumatic spinal cord injury from January 2016 to December 2020 at King Abdulaziz Medical City (KAMC), Riyadh, Saudi Arabia. KAMC is a tertiary care hospital that serves a population of the National Guard soldiers and their dependents in Riyadh. The inclusion criterion for this study is all patients 18 years and older who were surgically treated for nontraumatic spinal cord injury from 2016 to 2020 with a minimum follow-up of 12 months postoperatively. Moreover, any traumatic case of spinal cord injury and nontraumatic spinal cord injuries that were treated nonsurgically will be excluded.

### Data Collection

All patients who had a nontraumatic spinal cord injury will be reviewed; thus, the sampling technique to be used is total population inclusion. The data will be collected from patients' files using the BestCare system or documented files to retrospectively identify all patients who had a nontraumatic spinal cord injury during 2016 to 2020 at KAMC.

The variables in the Excel data sheet will include serial numbers, age, smoking status, sex, body mass index, comorbidities, previous surgery, name of procedure, odds ratio (OR) time in minutes, length of stay, level of injury, American Spinal Injury Association (ASIA) score preoperatively and postoperatively, ASA score, need for spinal cord injury rehabilitation postoperatively, unplanned 90-day readmission and its causes, complications, and 1-year survival.

### Statistical Analysis

Sex, comorbidities, medical history, presenting symptoms, diagnosis, and level of injury were reported as frequency and percentage. The variables were compared between groups who had postsurgery improvement/no change by using the chi square test. *P*-value less than 0.05 was considered significant. Age, body mass index (BMI), and ASA scores were compared by using the Wilcoxon rank sum test. Odds ratio time and blood loss were reported as mean and standard deviation. Length of stay was reported as median and interquartile range. Odds ratio time, blood loss, and length of stay were compared using the Kruskal-Wallis test. Logistic regression was used to identify the predictors of postsurgery improvement. The dependent variable was post-ASIA score level E versus others. The independent variables (age, BMI, sex, diagnosis, and injury level) were identified by bivariate analysis and clinical judgment. The results were reported as ORs along with their 95% confidence intervals (95% CI). The discriminatory power and calibration of the multivariate models were evaluated using concordance (C) statistics. All the analyses were conducted using SAS software, version 9.4.

## Results

A total of 164 patients with nontraumatic spinal cord injuries at KAMC were recorded in this study. Patients' demographics and clinical characteristics are presented in Table 1. Among the 164 patients, 68 (41.46%) were male, and 96 (58.54%) were female. The average age of the participants was 61 ± 13.01 years. Moreover, the mean BMI was shown to be 30.50. Regarding smoking, only 14 patients (8.54%) were smokers. In addition, 134 patients (81.71%) were recorded to have comorbidities, with hypertension (84, 51.22%) being the highest, followed by diabetes (81, 49.39%) and dyslipidemia (57, 34.76%). Most of the patients had an ASA score of 2 ± 0.65. As to patient presentation, back pain was the most frequent report, followed by numbness and finally the inability to walk with 86 (52.44%), 68 (41.46%), and 39 (24%) patients, respectively.

**Table 1:** Demographics

Sex	Male (%)	Female (%)
	68 (41%)	96 (59%)

	Yes (%)	No (%)
Smoking status	14 (9%)	150 (91%)
Comorbidities	134 (82%)	30 (18%)
Hypertension	84 (51%)	80 (49%)
Diabetes	81 (49%)	83 (51%)
Dyslipidemia	57 (35%)	107 (65%)
Hypothyroidism	17 (10%)	147 (90%)
Asthma	14 (9%)	150 (91%)
Ischemic heart disease	13 (8%)	151 (92%)
Osteoarthritis	7 (4%)	157 (96%)
Arrythmias	3 (2%)	162 (98%)
Rheumatoid arthritis	2 (1%)	162 (99%)
Previous strokes	2 (1%)	162 (99%)
Osteoporosis	1 (1%)	163 (99%)
Symptoms on admission		
Back pain	86 (52%)	78 (48%)
Numbness	68 (41%)	96 (59%)
Inability to walk	39 (24%)	125 (76%)
Neck pain	25 (15%)	139 (85%)
Radiculopathy	37 (23%)	127 (77%)
Urinary incontinence	18 (11%)	146 (89%)
Bowel incontinence	4 (2%)	160 (98%)

**Figure 1:**
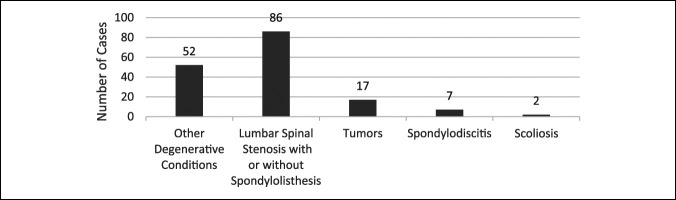
A bar chart demonstrating the number of cases for each nontraumatic spinal cord injury etiology.

Lumbar spine injuries accounted for 81 cases (49%), cervical injuries for 58 (35.37%), and thoracic injuries for 25 (15.24%). Regarding the diagnosis, as shown in Figure 1, degenerative spine conditions (such as lumbar spinal stenosis with or without degenerative spondylolisthesis, cervical spondylosis with myelopathy, thoracic myelopathy, and disk herniation) were the most common conditions in the case series with 138 patients (84%). The most common cause among the degenerative conditions was lumbar spinal stenosis with or without spondylolisthesis accounting for 86 patients (52.44%) of the population. Tumors were reported to be found in 17 (10.37%) while spondylodiscitis and scoliosis/kyphosis were the least observed among the patients with 7 (4.27%) and 2 (1.22%), respectively. In addition, thoracic and lumbar laminectomy/fusion (83, 50.61%) was the most conducted procedure, and anterior/posterior cervical corpectomy and fusion were the least conducted procedures (16, 9.76%), as summarized in Table 2. Patients treated with anterior cervical diskectomy and fusion had the lowest mean OR time (272 minutes), blood loss (201 mL), and length of stay (11.8 days) while segmental internal fixation/spinal instrumentation recorded the longest mean OR time (338 mins) and length of stay (38 days) and thoracic/lumbar laminectomy recorded the highest blood loss (409 mL).

**Table 2:** Procedure, OR Time, Blood Loss, and LOS

Procedure	N	Variable	Minimum	Maximum	Mean	SD	Median	Quartile Range
Anterior cervical diskectomy and fusion	39	OR time	125	480	273	80	264	64
		Blood loss	50	1000	201	254	100	100
		LOS	3	39	12	10	7	8
Anterior/posterior cervical corpectomy and fusion	16	OR time	20	502	333	114	360	71
		Blood loss	100	1000	319	217	300	150
		LOS	11	58	32	14	30	22
Thoracic/lumbar laminectomy/fusion	83	OR time	35	660	330	114	360	120
		Blood loss	0	1500	409	271	400	300
		LOS	3	75	20	17	13	14
Segmental internal fixation/spinal instrumentation	26	OR time	78	660	339	147	300	214
		Blood loss	50	1000	383	214	400	300
		LOS	7	279	38	60	16	25

OR = odds ratio, LOS = length of stay

The overall recorded complication rate in 164 patients was 17% (n = 29/164). Thoracic and lumbar laminectomy/fusion was the most recorded surgery to have an associated complication following it (7.9%, n = 13/164). Furthermore, of the 95 patients who had a complete neurological recovery, 11 developed a complication during hospital stay (12%, n = 11/95). Neuropathic pain was the most recorded concern (9%, n = 9/95), followed by urinary tract infection (3%, n = 3/95). In addition, urinary incontinence and constipation were recorded in 2% (n = 2/95) and 1% (n = 1/95), respectively. Contrarily, 18 of 69 patients who had a poor outcome of the ASIA score developed a complication (26%, n = 18/69). The most common complication seen was UTI with 13% (n = 9/69) and then neuropathic pain with 11% (n = 8/69).

A total of 129 patients had an ASIA score of “D” at admission, and 66% (n = 85/129) had a full recovery with a score of “E” at the last follow-up. Twenty-seven patients had a score of “C,” and 93% (n = 25/27) had an improvement at the last follow-up, with 17 patients with a score of “D” and 8 with a score of “E.” Two patients had a score of “B,” and both patients showed a score of “D” and “E” at the last follow-up. Five patients had an ASIA score of “A” at admission, and four had an improvement at the last follow-up (Table 3).

**Table 3:** American Spinal Injury Association Score

Preoperative ASIA Score	ASIA Score at Last Follow-Up					
Scores	A	B	C	D	E	Total
A	1 (0.6%)	1 (0.6%)	2 (1.2%)	0	1 (0.6%)	6 (3%)
B	0	0	0	1 (0.6%)	1 (0.6%)	2 (1.2%)
C	0	0	2 (1.2%)	17 (10.4%)	8 (4.9%)	27 (16.5%)
D	0	0	0	45 (27%)	85 (52%)	130 (79%)
Total	1 (0.6%)	1 (0.6%)	4 (2.4%)	63 (38%)	96 (58.2%)	164 (100%)

Table 4 presents that of the 164 patients, 95 (57.93%) had a full recovery whereas 69 (42.07%) had a poor ASIA score outcome.tvmjline 0pt Moreover, 76.8% of the patients who achieved full neurological recovery had at least one comorbidity (n = 73/95). Hypertension was the most common recorded comorbidity (50.5%, n = 48/95), followed by diabetes (44.2%, n = 42/95). Contrarily, patients with a poor outcome recorded higher comorbidity rates (86.9%, n = 60/69). Diabetes was the most common comorbidity (55%, n = 38/69), followed by hypertension (50.7%, n = 35/69). Few variables were markedly associated with a poor ASIA score outcome at the last follow-up. Patients who presented with urinary incontinence and/or bowel incontinence on admission had a significantly worse ASIA score at the last follow-up (55%, n = 10/18. *P* = 0.0075; 75%, n = 3/4, *P* = 0.0380). Degenerative spondylotic cervical myelopathy and degenerative thoracic myelopathy recorded a worse ASIA score than any other pathology (22.5%, n = 37/164, *P* = 0.0082). Furthermore, patients with a cervical or lumbar level of injury had shown nearly four times the improvement compared with the thoracic injury level (*P* = 0.049, *P* = 0.031).

**Table 4:** Analysis of Maximum Likelihood Estimates

Parameter	Estimate	Standard Error	Wald Chi Square	Pr > Chi Square	
Intercept	0.8366	1.5319	0.2983	0.5850	
Age	0.0150	0.0158	0.8976	0.3434	
BMI	−0.0275	0.0323	0.7270	0.3938	
Sex					
Female	0.0441	0.4019	0.0121	0.9126	
Diagnosis					
Degenerative	−1.7701	0.6691	6.9994	0.0082	
Herniated disk	−0.2190	0.6910	0.1004	0.7513	
Others	0.2512	0.7292	0.1186	0.7305	
Injury level					
Cervical	1.3483	0.6732	4.0111	0.0452	
Lumbar	1.2792	0.5806	4.8535	0.0276	

	Improved	Not Changed	Total	Chi Square (Value)	Chi Square (prob)
Urinary incontinence				7.1544	0.0075
No	109	37	146		
Yes	8	10	18		
Bowel incontinence				4.3065	0.0380
No	116	44	160		
Yes	1	3	4		

BMI = body mass index

## Discussion

Nontraumatic spinal cord injury has been described in a few previous papers with an estimated incidence of 5.1 to 80 per million population per year.^10^ Our study showed that among the 164 patients, a greater number of female patients were affected by NTSCIs than male patients. Contrarily, other studies showed that both sexes were affected equally.^6,11^ In addition, the mean age in our study was 61 years, which is a similar finding in many previous studies with a mean age between 40 and 62 years.^7,11,12^ All in all, there was no statistical significance in sex and age with the neurological outcome. Furthermore, most of the patients presented with back pain as the most frequent report, followed by numbness and finally the inability to walk. However, patients presenting with urinary or bowel incontinence had poor prognosis statistically in neurological recovery at the final follow-up, and one possible explanation may be related to relatively late presentation of these patients and the severity and advanced stage of the NTSCI. Comparably, a study showed that occasionally sphincter dysfunction was markedly associated with NTSCI; however, back pain, tendon reflexes, and muscle strength did not differ statistically.^7^

In our study, the most frequent underlying pathologies were degenerative conditions. However, a study demonstrated that almost half of all NTSCIs were caused by degenerative disorders, followed by neoplastic conditions.^13^ Similarly, two studies, one in Ireland only and another cited in Western Europe, showed that degenerative conditions were the most frequent and tumors the second most common.^1,13^ A Canadian health database also found the most common etiology of NTSCI to be degenerative conditions.^14^ On the contrary, previous studies reported tumors to be the most common etiology in their series.^8,15^ A systematic review of NTSCI in sub-Saharan Africa noted that the most common etiologies were Pott disease and metastases conditions.^16^ All in all, our results showed that patients with degenerative conditions had a worse improvement outcome in comparison with other causes which had a notable improvement on follow-up. One possible explanation is the gradual, long-lasting insidious onset of the nontraumatic spinal cord injuries due to degenerative pathologies while patients with NTSCI due to tumor or infectious underlying causes often present earlier because of the systemic disease, other associated symptoms, and pain. Regarding the level of injury, we found that injuries to the cervical or lumbar spine have shown four times the neurological improvement compared with those in the thoracic spine. It may be because the thoracic spine has fewer arteries than in other areas (average, one to four), poor collateral potential, and no direct communication between anterior and posterior systems. These factors are thought to be responsible for the sensitivity of this region to any insult and the less recovery rate of the thoracic injury compared with the other areas.^15^ Unfortunately, we were unable to find a study that supports our finding and investigates the improvement among the spinal levels in NTSCI. It may be because of studies usually focusing on the etiology rather than the level of the injury.

NTSCI often presents with different spectrums of neurological deficits. Our findings are consistent with previously published literature that found ASIA score D as the most frequently recorded score. A study conducted on 129 cases in Ireland reported that 56% of the cases were ASIA score D.^13^ Another study in the Netherlands reported that 67.2% of the cases were ASIA score D, followed by score C in 14.3%.^11^ Furthermore, 95 patients (57.93%) have shown full neurological recovery in their admission ASIA score versus the last follow-up score. This is similar to another study that concluded that 58% of the patients had notable neurological recovery.^16^ A different study investigated the improvement rate of patients with a score of A, B, and C and found that 25% of the patients had improved with at least one score,^5^ whereas, in our study, 91.1% of the patients with A, B, and C scores have shown improvement with at least one score (n = 31/34). None of our patients have shown a worse ASIA score compared with their preoperative scores. Contrarily, a study recorded a worse functional status in 2.4% of their patients during inpatient rehabilitation.^11^

The main limitation of our study is being a single-center retrospective study with a relatively small sample size, particularly the sample size for each etiology, and we did not include the nonsurgical cases as a control group. Another limitation is related to not being able to have a standard surgical or rehabilitation treatment method in each group. All in all, and as far as we are aware, this is the first study to discuss the aspects and patterns of NTSCI in our region.

## Conclusion

Surgically treated NTSCI showed good neurological recovery with a low complication rate. Additional multicenter and prospective studies are warranted to identify the true prognostic factors in NTSCI.
